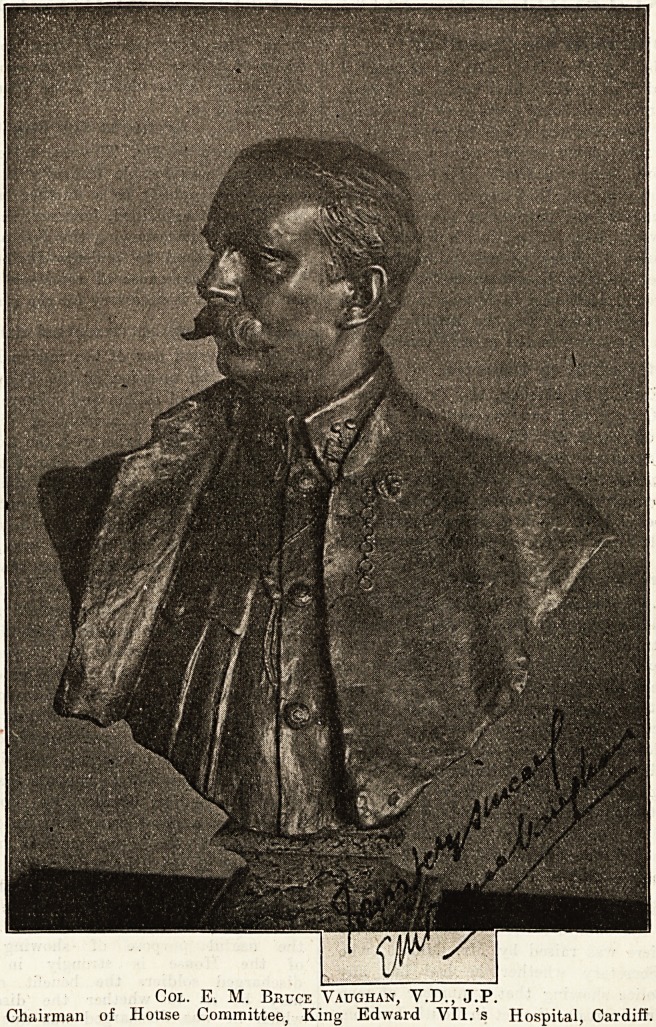# Colonel Bruce Vaughan's Assured Victory

**Published:** 1916-02-26

**Authors:** 


### THE WELSH NATIONAL SCHOOL OF MEDICINE.

### Colonel Bruce Vaughan's Assured Victory.

THURSDAY, the 17th instant, was a great day in Cardiff for all who take an interest in its medical institutions; that is, for every patriotic Welshman throughout the Principality. On this day, in the presence of the leaders and workers in philanthropic endeavour, at King Edward VII.'s Hospital, Cardiff, Lord Aberdare unveiled the portrait of the late King Edward VII. by Miss Lindsay Williamson, declared the new wing of the hospital open, with accommodation for 150 beds, and unveiled the bust of Colonel E. M. Bruce Vaughan, by Sir Goscombe John. General H. H. Lee presided. In the course of his remarks Lord Aberdare complimented Miss Williamson on her work, and did full justice to the generosity of Mrs. Nixon in presenting the portrait to the institution, whilst he declared it was most appropriate that the hospital should have a bust of Colonel Bruce Vaughan, who had done so much for it.

COL. E. M. BRUCE VAUGHAN, V.D., J.P.
Chairman of House Committee, King Edward VII.'s Hospital, Cardiff.

### ITS ONLY ENEMY THE GOVERNMENT.

Three remarkable points must be emphasised in regard to this school. First, it would have been practically built by this time but for the Treasury's action in stopping the erection of adequate buildings which would help to make it one of the most complete and efficient, as it should prove to be one of the most interesting, medical schools in the United Kingdom. Next, Wales is the only portion of the United Kingdom that has not even ONE medical school within its area. Finally, the completion of the school has been rendered possible through Sir William James Thomas' generosity in making the most princely gift ever offered to Wales by any single individual throughout her history. Since 1880 universities have been established at Manchester, Liverpool, Leeds, Sheffield, Birmingham, and Bristol, all of which have medical schools. Scotland and Ireland have each five bodies granting medical diplomas and nine complete schools of medicine. Wales alone has no national medical school, and is still without one, despite the fact that two years ago, following the splendid development of the King Edward VII 's Hospital, Cardiff, and the encouraging growth in the number of medical students attending there, Sir William Thomas' generosity provided the means at once to create and establish there a national school of medicine. All financial questions were disposed of finally, and the injustice and unwisdom of encouraging further delay became still more manifest when Sir William Thomas further promised to maintain the medical school buildings until such time as the Treasury were able to make an adequate grant. This left only a relatively small sum to be provided by the Treasury to defray the expenditure for such administrative and maintenance charges as were required and would be governed by the number of students entering.

In 1915, owing to the widespread belief that Wales was to have a full-time medical school in Cardiff, the students' entries were larger there than at Oxford, Liverpool, Birmingham, Manchester, and most of the London schools. Why, then, has the Government so far prevented the gratification of the wish of the Welsh people to have a national medical school, the very existence of which has a unique importance at the present moment owing to the serious shortage in doctors with which the country is threatened? It is for the Government to say, though the reason cannot be based on a wise policy or any principle of justice. We are therefore hopeful that an end may be at once put to the Treasury's past action, and the medical school buildings be allowed to be proceeded with with all possible dispatch.

## Figures and Tables

**Figure f1:**